# Supplementary material for: Effects of an App-Based Intervention to Improve Awareness and Usage of Early Childhood Intervention Services During the COVID-19 Pandemic: Randomized Controlled Trial of the CoronabaBY Study from Germany
Source: Healthcare (Basel). 2025 Aug 14;13(16):2000. doi: 10.3390/healthcare13162000 (PMC12386006; doi:10.3390/healthcare13162000)
Supplement: Supplementary file 1 [file healthcare-13-02000-s001.zip › Supplement_S2_questionnaire_ECI.pdf]

Questionnaire: Evaluation of Early Childhood Intervention services

1. When you have a baby or toddler, it sometimes feels like things are getting out of hand. Do you know that there are free, non-binding support services for young families for such situations (so-called 'Frühe Hilfen' – this includes, for example, parenting advice, crybaby advice, baby/child groups)?
   _yes *(-> continue with 1.b.)*
   _no *(-> continue with 2.)*

   1. b. How do you know 'Frühe Hilfen'?

   _through my gynecologist
   _through our pediatrician
   _through the KoKi
   _through an internet search
   _through family/friends/acquaintances
   _through posters/flyers
   _through a so-called 'welcome visit'
   -> only for post-test and follow-up: _through this app ('my pediatrician')
   _other: ________________ (open)

2. Here, you find a selection of support services that belong to 'Frühe Hilfen'. Which of these services have you perhaps already used (multiple answers possible)?

   -> alternative wording for post-test and follow-up: Has your family accessed any of the following services of 'Frühe Hilfen' since the last survey stage? (multiple answers possible)

   Support from:

   _the KoKi
   _a family midwife/family pediatric nurse
   _a volunteer (e.g., family mentor)
   _a parenting counseling center/family counseling center
   _a pregnancy counseling center
   _outreach services (to promote bonding), such as STEEP
   _developmental psychological counseling, PeKiP, Safe
   _cry baby counseling/cry baby outpatient center
   _baby/child groups and other family education services
   _other:________________ (open)
   _none of the above services or anything similar (*filter: the questionnaire ends with this option*)

3. How did you become aware of this service/these services?

   _through my gynecologist
   _through our pediatrician
   _through the KoKi
   _through an internet search
   _through family/friends/acquaintances
   _through posters/flyers
   _through a so-called 'welcome visit'

_other: ________________ (open)

4. Who reffered you to this service/these services? (multiple answers possible)

   _my gynecologist
   _our pediatrician
   _the KoKi
   _I made the contact myself

5. How quickly was the referral to the support service(s) possible?

   _immediate referral to support service(s) (no waiting time)
   _timely referral to support service(s) (a few days' waiting time)
   _delayed referral to support service(s) (a few weeks' waiting time)
   _significantly delayed referral to support service(s) (several weeks' waiting time)

6. How well did the support services provided fit your needs? (on a scale from 1 'very well' to 5 'not at all')

   ☐ 1 ☐ 2 ☐ 3 ☐ 4 ☐ 5
   very well ——— not at all

7. How helpful were the support services provided to you? (on a scale from 1 'very helpful' to 5 'not helpful at all')

   ☐ 1 ☐ 2 ☐ 3 ☐ 4 ☐ 5
   very helpful ——— not helpful at all

8. Over what period of time did you receive the support service(s)?

   _ once
   _up to 4 weeks irregularly
   _up to 4 weeks regularly
   _over several months irregularly
   _over several months regularly
   _over one year irregularly
   _over one year regularly
   _more than one year

9. Are you currently receiving the support service(s) mentioned above?

   _yes, still currently
   _no, completed
