# Supplementary figures and images for: Effects of an App-Based Intervention to Improve Awareness and Usage of Early Childhood Intervention Services During the COVID-19 Pandemic: Randomized Controlled Trial of the CoronabaBY Study from Germany
Source: Healthcare (Basel). 2025 Aug 14;13(16):2000. doi: 10.3390/healthcare13162000 (PMC12386006; doi:10.3390/healthcare13162000)

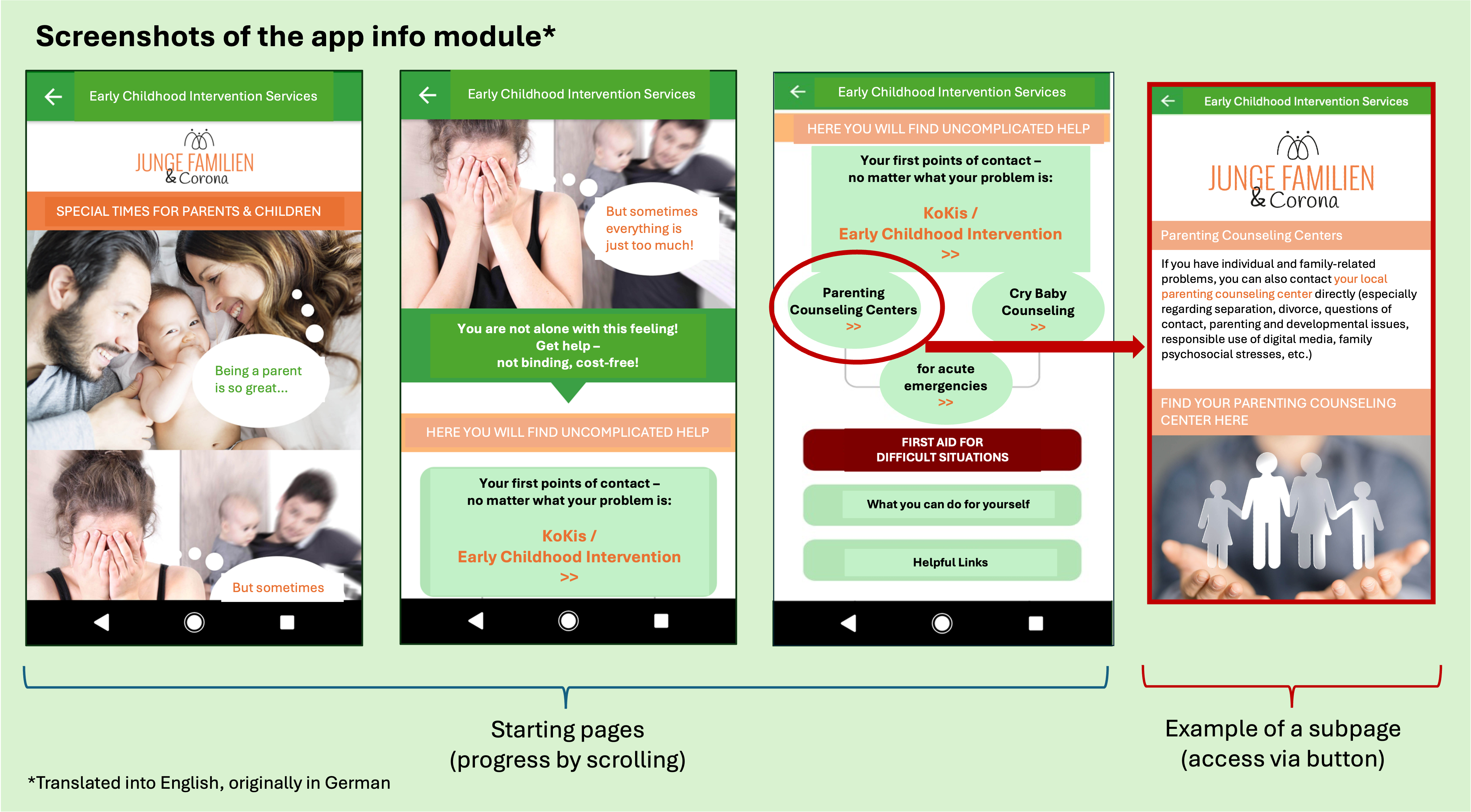

Supplement: Supplementary file 1 [file healthcare-13-02000-s001.zip › Supplement_S1_screenshots_app_module.png]
